# The symptoms and interval of Omicron SARS-CoV-2 reinfection among healthcare workers in a hospital of Southern China: a cross-sectional study

**DOI:** 10.1186/s12879-024-09221-3

**Published:** 2024-03-27

**Authors:** Xiaoju Ma, Zheng Wang, Youpeng Chen, Zhanjie Li

**Affiliations:** 1 Department of Hospital Acquired Infection Control and Public Health Management, The Seventh Affiliated Hospital, Sun Yat-sen University, No.628 Zhenyuan Road, 518107 Shenzhen, Guangdong China; 2 Department of Infectious Diseases, The Seventh Affiliated Hospital, Sun Yat-sen University, Shenzhen, Guangdong China; 3 Department of Infection Control, The First Affiliated Hospital of Nanjing Medical University, No. 300 Guangzhou Road, 210029 Nanjing, Jiangsu China

**Keywords:** SARS-CoV-2 infection, Reinfection, COVID-19, Omicron, Healthcare workers

## Abstract

**Background:**

The prevalence and distinction between first Severe acute respiratory syndrome coronavirus 2 (SARS-CoV-2) infection and reinfection with the Omicron variant among healthcare workers (HCWs) remain unclear.

**Methods:**

A cross-sectional study was conducted at a hospital in Southern China. The study included 262 HCWs who were infected with SARS-CoV-2 between April and June 2023, with 101 cases of first infection and 161 ones of reinfection. Student’s t-test, Analysis of Variance (ANOVA), and Mann-Whitney U tests were used based on the distribution of quantitative variables. Pearson’s chi-square and Fisher’s exact tests were used based on the expected frequencies of categorical variables.

**Results:**

The reinfection rate among HCWs was 11.5% (161/1406). The majority of the infected HCWs were female (212/262, 80.9%, first infection vs. reinfection: 76.2% vs. 83.9%). The nursing staff, had the highest percentage of SARS-CoV-2 infection (42.0%), especially of its reinfection (47.8%). Out of the 262 infected individuals, 257 had received SARS-CoV-2 vaccination, primarily inactivated vaccines (243/257, 91.1%). The first infection group, which received four doses (24, 23.8%), was significantly higher than that in the reinfection group (6, 3.7%) (*P* < 0.001). The proportion of asymptomatic infections among HCWs in the two groups was 1.0% and 1.2%. The main symptoms during the first infection and reinfection were fever (83.2% and 50.9%) and sore throat (78.2% and 77.0%). There were significant differences in the prevalence of fever (83.2% vs. 50.9%), rhinorrhea (45.5% vs. 60.9%) and myalgia (56.4% vs. 37.9%) between the first infection and reinfection (*P* < 0.05). The average interval for SARS-CoV-2 reinfection was 149.9 (range: 114–182, SD = 11.9) days. Notably, physicians had the shortest average interval of 142.8 (8.8) days, while management and administrative staff had the longest average interval of 153.8 (13.5) days.

**Conclusions:**

Although the symptoms of HCWs during reinfection with SARS-CoV-2 were milder, the high reinfection rate and short interval between infections indicate the need to enhance monitoring and protective measures for HCWs during the epidemic.

**Supplementary Information:**

The online version contains supplementary material available at 10.1186/s12879-024-09221-3.

## Background

Severe acute respiratory syndrome coronavirus 2 (SARS-CoV‐2), the cause of coronavirus disease 2019 (COVID-19), has rapidly spread worldwide since December 2019, and COVID-19 pandemic has been ongoing for over three years [1]. With the widespread and continuous evolution of SARS-CoV-2, the World Health Organization (WHO) has identified and designated multiple variants of concern (VOCs) and variants of interest (VOIs), including Alpha, Beta, Gamma, Delta, and Omicron [2]. Omicron, first identified on November 2021 [3], has emerged as the most predominant mutated variant since 2022. This may be attributed to its higher transmissibility, decreased vaccine effectiveness, or reduced effectiveness of the public health measures [4, 5]. On December 7, 2022, China adjusted and optimised its prevention and control strategies in light of the current status of the COVID-19 epidemic [6]. As a result, the number of cases increased rapidly [7]. According to the Chinese Center for Disease Control and Prevention (China CDC), Omicron BA.5.2 and BF.7 were the dominant variants from December 2022 to February 2023, while XBB was the most prevalent variant during the second outbreak between April 2023 to June 2023 [8]. The immune evasion capabilities of Omicron sublineages BQ.1.1 and XBB have been reported to be greater than those of earlier Omicron variants (like BA.5 and BA.2) [9, 10]. These studies suggest that the sublineages of Omicron are becoming increasingly evasive to antibodies and more transmissible.

Although SARS-CoV‐2 could lead to various symptoms occurring in a wide range of systems [11], Omicron is associated with a decrease in disease severity. This is because the viral load is higher in the upper airway, specifically in the nose, windpipe, and throat [5, 12–15]. Researchers have reported that the most common symptoms of Omicron infection include sore throat, cough, runny nose, congestion, and fatigue [11, 16]. In addition, studies have shown that fever and myalgia are also the most frequent symptoms in Omicron-infected patients from China [17, 18]. These serve as a reminder that more research has to be done on the symptoms of Omicron infection.

Omicron infection develops milder symptoms, but its reinfection rate is higher than that of the earlier strains [19] Before November 2021, the global reinfection rate is low to 5% [15, 20–22]. After the emergence of the Omicron variant, this rate is over 10% [22–25]. The interval between primary pre-omicron infection and pre-omicron or omicron reinfection was reported to range from 45 to 672 days [26, 27]. Most of the reinfected patients are asymptomatic, with milder symptoms or symptoms similar to those of the first infection [20, 23]. However, the interval and symptoms of Omicron reinfection compared to the first Omicron infection remain poorly studied.

Healthcare workers (HCWs) have been exposed to a higher risk of SARS-CoV-2 infection than the general population, especially those assigned to job tasks involving direct or close contact with COVID-19 patients [28, 29]. Omicron epidemic data indicate that HCWs accounted for 7% of the infection cases [12]. A Systematic Review and Meta-Analysis suggested that HCWs have a higher rate of reinfection [30]. However, there is no clarity regarding the prevalence and distinction between first SARS-CoV-2 infection and reinfection with the Omicron variant among HCWs.

Overall, this study aimed to analyze the epidemiological characteristics of first SARS-CoV-2 infection and reinfection associated with the emergence of the Omicron variant in HCWs.

## Methods

### Study design

**Fig. 1:**
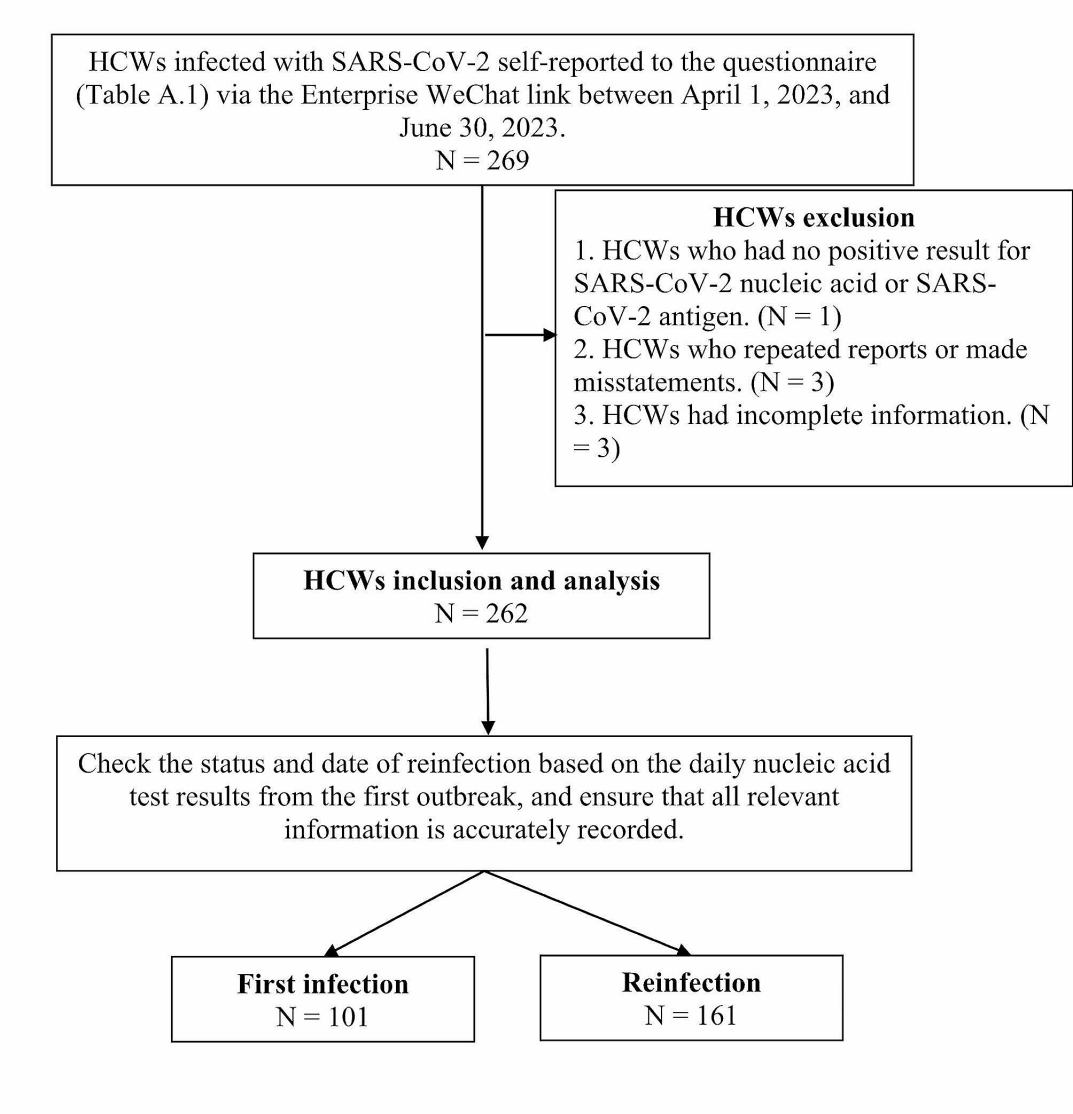
Flowchart of HCWs exclusion and inclusion

From April 1 to June 30, 2023, we created an Enterprise WeChat link for HCWs to self-report SARS-CoV-2 infections and symptoms (Table A.1) from the Seventh Affiliated Hospital, Sun Yat-sen University. A total of 269 HCWs participated in the survey. For this analysis, we excluded cases with repeated reports, incomplete personal information, and those without confirmation of nucleic acid amplification tests or antigen self-tests (*n* = 7). The flowchart of this study is displayed in Fig. 1. Prior to this study, a total of 1406 members of the HCWs had been infected once, and 161 in this study had a secondary infection, giving a reinfection rate of 11.5% (161/1406). All the work was approved by the biomedical research ethics committee, The Seventh Affiliated Hospital, Sun Yat-sen University. The ethics approval number is KY-2024-050-01.

### Case definition

SARS-CoV-2 infection was confirmed when real-time fluorescence SARS-CoV-2 reverse transcription-polymerase chain reaction (RT-PCR) or SARS-CoV-2 antigen was positive. First infection was defined as a first occurrence of SARS-CoV-2 infection. After infection with SARS-CoV-2, both humoral and cellular immunity make it less likely for a patient to be reinfected. However, with the decline in antibody titers, specifically for SARS-CoV-2, some earlier studies reported a rapid waning of antibody responses, diminishing after 90 days. In addition, low viral loads that do not represent replicative virus could lead to a recurrence of positive (re-positive) nucleic acid detection within 90 days. For the above reasons, many recent studies [31, 32] define reinfection as occurring after more than 90 days. Therefore, in this study, reinfection was defined as a second occurrence of SARS-CoV-2 infection, which occurred at least 90 days after the first infection [33].

SARS-CoV-2 RT-PCR was conducted using a detection kit (Daan Gene, Guangzhou, China). RNA expressed from the nucleocapsid (N) and ORF1ab genes of SARS-CoV-2 was detected from oropharyngeal swabs. If the cycle threshold (Ct) value of the test sample in the FAM and VIC channels is < 35 and there is a significant amplification curve, the sample can be judged as positive. If the Ct value is only ≤ 35 in either the FAM or VIC channel, and there is no amplification curve in the other channel, the result needs to be reexamined. If the reexamined result is consistent with the previous one, the sample could be judged to be positive for SARS-CoV-2.

To detect the SARS-CoV-2 antigen, the SARS-CoV-2 antigen kit (for self-testing) based on the colloidal gold method (Wondfo, Guangzhou, China) was applied. The SARS-CoV-2 antigen kit is a membrane-based immunoassay designed to detect SARS-CoV-2 nucleocapsid protein antigens in human respiratory samples from oropharyngeal, nasopharyngeal, and nasal swabs, etc. A colored line will appear in the test line region if the specimen contains SARS-CoV-2 antigens.

### Information collection

The information of the study participants was collected, including age, gender, type of employment [physicians, nursing staff, other health-assisting occupations (such as radiologists, pathologists, sonographers, anesthetists, medical technicians and pharmacists), management and administrative staff], classification of medical personnel [Considering different specialties/working departments can have different exposure, medical personnel (physicians and nursing staffs) were classified into two categories based on the specialties and working department and the frequency of contact with patients. The department of medical staff category 1 include clinical departments (outpatient, emergency and inpatient wards), and the department of medical staff category 2 include medical technology support departments (such as radiology, ultrasound, physical examination, and operating room)], date of the infection, positive test results, symptoms, previous infection (yes or no), and time to the first infection through the self-reported questionnaire. We collected the SARS-CoV-2 vaccination records, including the type of vaccine, doses, and timing of each dose, from the vaccination system. Types of vaccines were classified as inactivated and non-inactivated based on the last dose. Non-inactivated vaccines include adenovirus vector vaccines, adenovirus vectors for inhalation, recombinant subunit vaccines, and mRNA vaccines. The doses of vaccines were divided into ≤ 2, 3 and 4 doses.

According to the Chinese Diagnosis and Treatment Protocol for Novel Coronavirus Infection (Trial version 10) [34], the typical symptoms of SARS-CoV-2 infection include fever (≥ 37.3℃), dry cough, nasal obstruction, rhinorrhea, sore throat, diarrhea, fatigue, myalgia, conjunctivitis, anosmia or dysgeusia and others. Others include headaches, dizziness, giddiness, expectoration, and vomiting.

The information of reinfections was cross-referenced with the surveillance lists for first infections.

### Quality control

In order to ensure the representativeness of our samples, we adopted the following strategies in our study: (1) We posted an Enterprise WeChat link in the hospital information system and the COVID-19 special contact person WeChat group, requesting that the COVID-19 special contact persons send the Enterprise WeChat link to their respective department WeChat groups to ensure coverage of employees from all departments and positions. All staff members who tested positive for SARS-CoV-2 antibodies or antigens were required to fill out and submit an electronic questionnaire. (2) To ensure the accuracy of self-reported cases, we required all reporting personnel to upload a photo of their SARS-CoV-2 antibody or antigen test certificate at the same time. (3) During the recruitment process, we set clear inclusion criteria, namely all staff members who tested positive for SARS-CoV-2 antibodies or antigens; we also set exclusion criteria, including repeated positive tests, inability to provide proof of SARS-CoV-2 antibody or antigen test, and incomplete personal information. (4) In order to detect asymptomatic carriers as much as possible and ensure the relative precision of infection time, we implemented mandatory weekly antibody testing for all staff at the hospital level, with symptomatic individuals being tested for antibodies or antigens at any time. In this study, the time of infection is determined based on the time when the antibody or antigen test result was positive.

To minimize selection bias as much as possible, we took the following measures: (a) During the recruitment process, we maintained close communication with the liaisons of each department to ensure they understood the importance of the study and encouraged staff participation. (b) We also established a dedicated research WeChat group, including the research team and liaisons from all departments, responsible for answering questions from potential participants and assisting them in completing the study process.

### Statistical analysis

Data were summarized as mean (standard deviation, SD), median (P25, P75), or frequencies (percentages, %), as appropriate. Student’s t-test, Analysis of Variance (ANOVA), and Mann-Whitney U tests were used based on the distribution of quantitative variables. Pearson’s chi-square and Fisher’s exact tests were used based on the expected frequencies of categorical variables. Statistical analyses were performed using Statistical Product and Service Solutions (SPSS), version 23.0 (IBM Corp., Armonk, NY, USA). Data graph was performed using GraphPad Prism 8.0 (San Diego, CA, USA). *P* < 0.05 was considered statistically significant.

## Results

### Frequency distribution of first infection and reinfection of SARS-CoV-2 in HCWs over time

A total of 262 HCWs were included in the study, of whom 101 (38.5%) were infected for the first time, and 161 (61.5%) were infected for the second time. In this hospital, there were 1406 HCWs infected with SARS-CoV-2 during the first wave of the outbreak (from December 2022 to January 2023). The rate of SARS-CoV-2 reinfection for HCWs in this wave was 11.5% (161/1406).

**Fig. 2:**
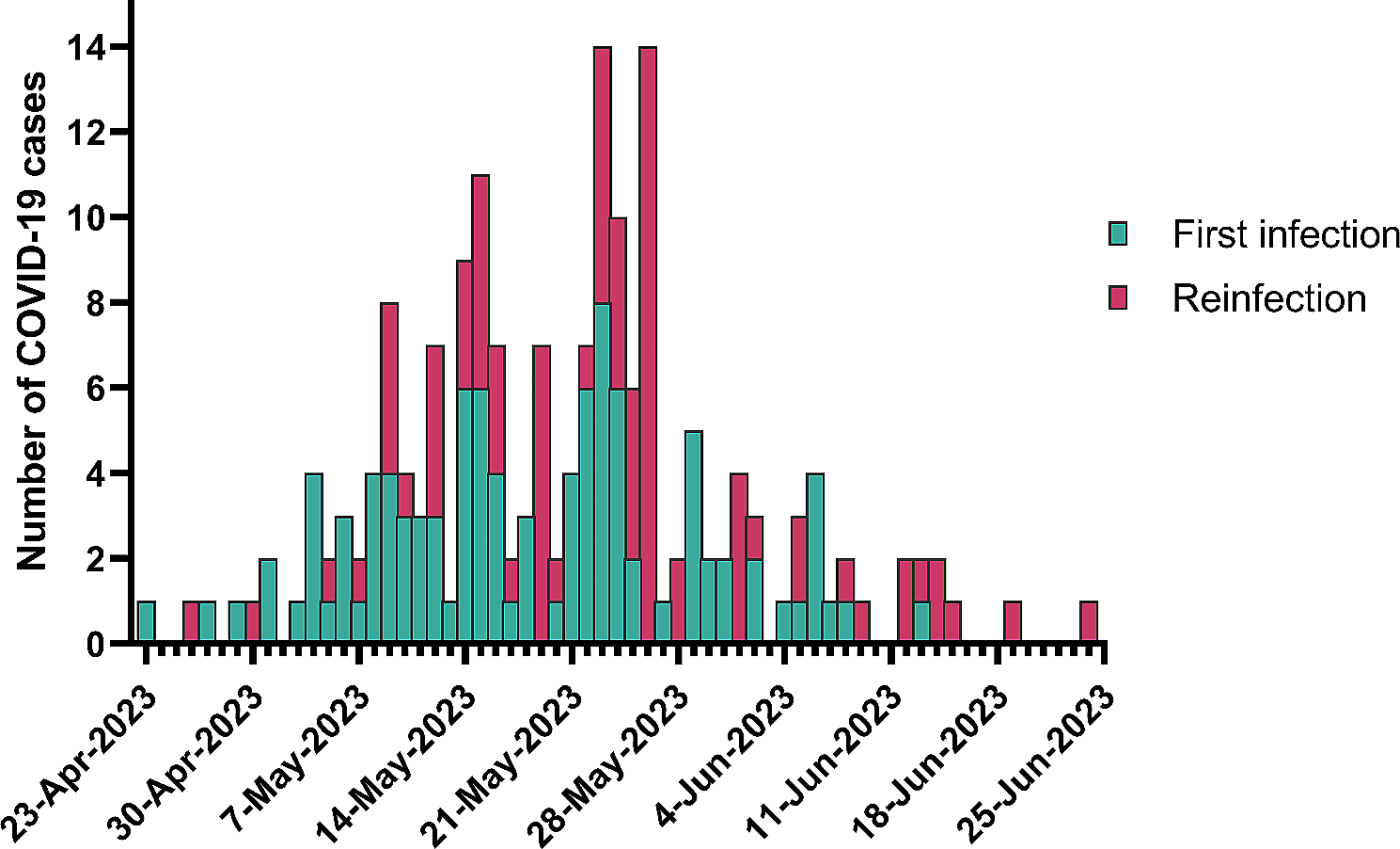
Frequency distribution of SARS-CoV-2 first infection and reinfection in HCWs over time, 1 April to 30 June, 2023

Figure 2 displays the frequency distribution of first SARS-CoV-2 infections and reinfections in HCWs between April 23, 2023, and June 24, 2023. It could be found that the infection frequency of the 262 cases increased rapidly from May 4, 2023, and it reached the peak on May 23 (14 cases) and May 26 (14 cases). After the peak, the fluctuation decreased. The first infection peak occurred on May 23rd with 8 cases, while the reinfection peak on May 26th with 14 cases.

### Characteristics and the SARS-CoV-2 vaccination of HCWs with SARS-CoV-2 infection

#### Characteristics

The majority of the HCWs who were infected with SARS-CoV-2 were female (212/262, 80.9%, first infection vs. reinfection: 76.2% vs. 83.9%). The median age of HCWs at the time of first SARS-CoV-2 infection was 29 years (P25, P75: 27, 32), while the median age of reinfection was 30 years (P25, P75: 27, 34). Among the various types of position, the nursing staff had the highest proportion of SARS-CoV-2 infection (110/262, 42.0%, first infection vs. reinfection: 32.7% vs. 47.8%), followed by other health-assisting occupations (64/262, 24.4%, first infection vs. reinfection: 26.7% vs. 23.0%). The baseline information (gender, age, type of employment, and classification of medical personnel) was not significantly different between the HCWs who were first infected and those who were reinfected (*P* > 0.05) (Table 1). Notably, no cases required hospital admission.

#### Analysis of the SARS-CoV-2 vaccination

Out of 262 healthcare workers (HCWs), 257 were vaccinated. In the first infection group, 81 individuals (81.0%) received the inactivated vaccine, while 153 individuals (97.5%) were in the reinfection group (*P* < 0.001). The first infection group, which received four doses (24, 23.8%), was significantly higher than that in the reinfection group (6, 3.7%) (*P* < 0.001). The interval between the latest dose and infection in the first group was 427.8 (205.3) days, while it was 536.2 (137.7) days for the reinfection group (*P* < 0.001) (Table 1).

**Table 1:** Characteristics of HCWs with first infection and reinfection of SARS-CoV-2

Characteristics	Total (*n* = 262)	First infection (*n* = 101)	Reinfection (*n* = 161)	*P*
Sex				
Female	212 (80.9)	77 (76.2)	135 (83.9)	0.127
Male	50 (19.1)	24 (23.8)	26 (16.1)	
Age, years	30 (27,33)	29 (27,32)	30 (27,34)	0.116
Age, years				
<30	128 (48.9)	52 (51.5)	76 (47.2)	0.500
≥30	134 (51.1)	49(48.5)	85 (52.8)	
Type of employment				
Physician	48 (18.3)	24 (23.8)	24 (14.9)	0.083
Nursing staff	110 (42.0)	33 (32.7)	77 (47.8)	
Other health-assisting occupations	64 (24.4)	27 (26.7)	37 (23.0)	
Management and administrative staff	40 (15.3)	17 (16.8)	23 (14.3)	
Classification of medical personnel				
Medical staff category 1	158(84.5)	57(79.2)	101(87.8)	0.111
Medical staff category 2	29(15.5)	15(20.8)	14(12.2)	
Type of vaccine				
Inactivated vaccine	234 (91.1)	81 (81.0)	153 (97.5)	< 0.001
Non-inactivated vaccine	23 (8.9)	19 (19.0)	4 (2.5)	
Doses of vaccine				
≤ 2	25(8.5)	7(6.9)	18(11.2)	< 0.001
3	207(79.0)	70(69.3)	137(85.1)	
4	30(11.5)	24(23.8)	6(3.7)	
Interval between the latest dose and infection	493.8 (175.2)	427.8 (205.3)	536.2 (137.7)	< 0.001

### Symptoms in HCWs infected and reinfected with SARS-CoV-2

**Fig. 3:**
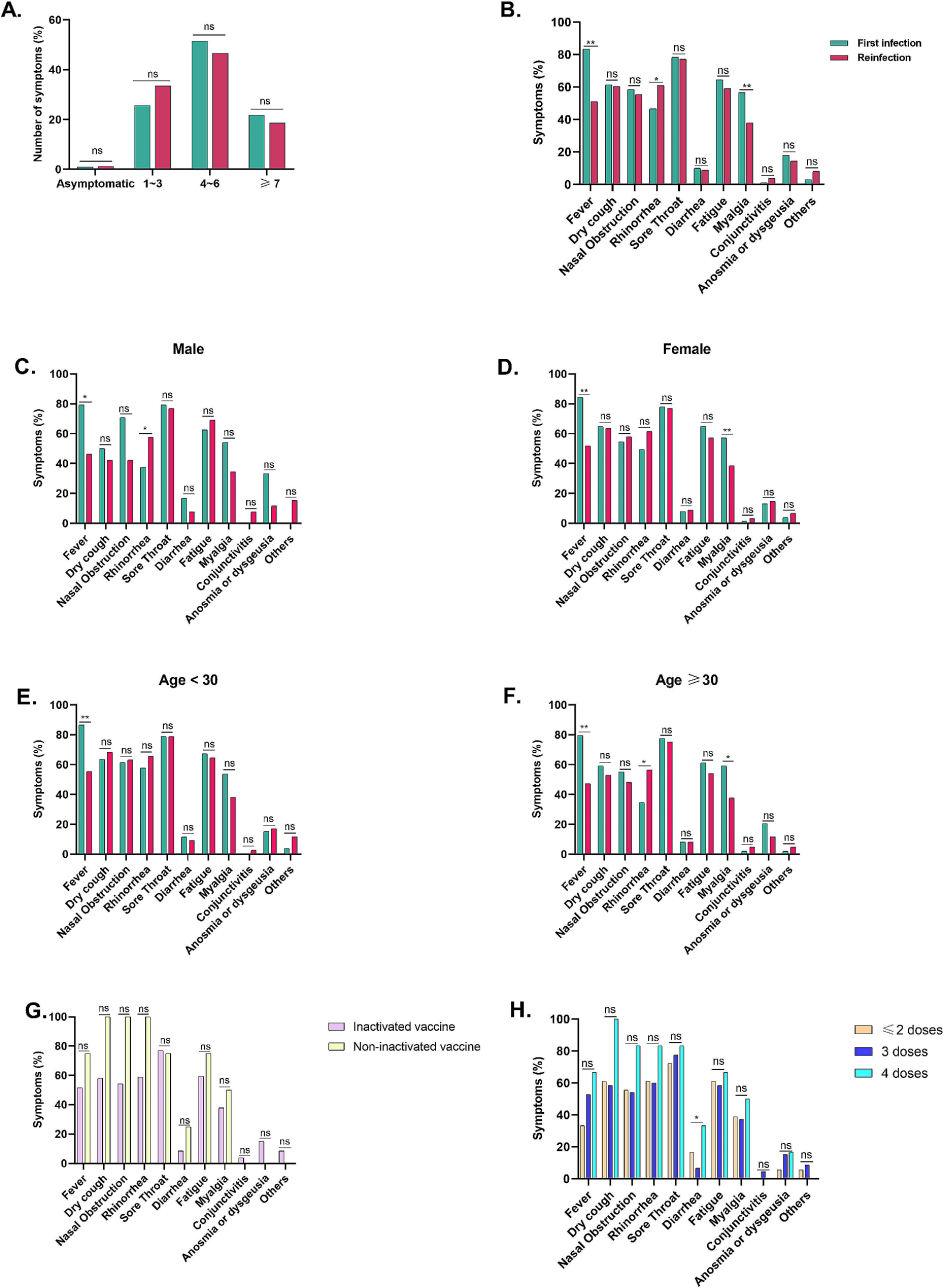
Symptoms in HCWs with first infection and reinfection of SARS-CoV-2. (**A**) The proportion of the number of symptoms in first-infected and reinfected HCWs. (**B**) The proportion of symptoms in the first infection versus reinfection among HCWs. (**C**) The proportion of symptoms in the first infection versus reinfection among male HCWs. (**D**) The proportion of symptoms in the first infection versus reinfection among female HCWs. (**E**) The proportion of symptoms in the first infection versus reinfection among HCWs age < 30 years. (**F**) The proportion of symptoms in the first infection versus reinfection among HCWs age ≥ 30 years. (**G**) The proportion of symptoms in the inactivated vaccine group versus non-inactivated vaccine group among reinfecion HCWs (161 cases). (**H**) The proportion of symptoms in the ≤ 2 doses, 3 doses and 4 doses of vaccination groups among reinfecion HCWs (161 cases). *P < 0.05 or ** *P* < 0.01 among the groups

In order to compare the number of symptoms between the fist infection and the reinfection groups, the symptoms were divided into four categories: asymptomatic, 1 ∼ 3, 4 ∼ 6, and ≥ 7 reported symptoms. The proportion of asymptomatic infections among HCWs in the two groups was 1.0% and 1.2%. The number of symptoms was dominated by 4 ∼ 6 symptoms in both groups (51.5% vs. 46.5%) (Fig. 3A). Fever (83.2%) and sore throat (78.2%) were mainly observed in HCWs with the first infection, whereas sore throat (77.0%) was more prevalent in HCWs reinfected with SARS-CoV-2. Among the first infected and reinfected HCWs, there were significant differences in the prevalence of fever (83.2% vs. 50.9%), rhinorrhea (45.5% vs. 60.9%), and myalgia (56.4% vs. 37.9%) (*P* < 0.05) (Fig. 3B). Additionally, there were statistically significant differences in fever between the two groups across different gender and age groups (all *P* < 0.05) (Fig. 3C ∼ 3 F). However, in both groups of first infected and reinfected individuals, rhinorrhea was only significant in males and in the age ≥ 30 years group, while myalgia was only significant in females and in the age ≥ 30 years group (all *P* < 0.05). These data indicate that the symptoms of reinfection are milder, and systemic symptoms, including fever and myalgia, are less severe than those of the first infection.

In order to explore the impact of SARS-CoV-2 vaccines on reinfection among HCWs, we categorized vaccine types and doses, and analyzed whether symptoms varied among the groups. There were no significant differences in symptoms between the HCWs who received the inactivated vaccine and those who received the non-inactivated vaccine (all *P* > 0.05). In three groups of ≤ 2 doses, 3 doses, and 4 doses of vaccination, diarrhea showed a significant difference (16.7% vs. 6.6% vs. 33.3%) (*P* < 0.05), while the other symptoms did not exhibit significant differences (*P* > 0.05). However, this may be related to the sample size of the vaccination group receiving four doses (6 cases).

### Interval of SARS-CoV-2 reinfection

**Fig. 4:**
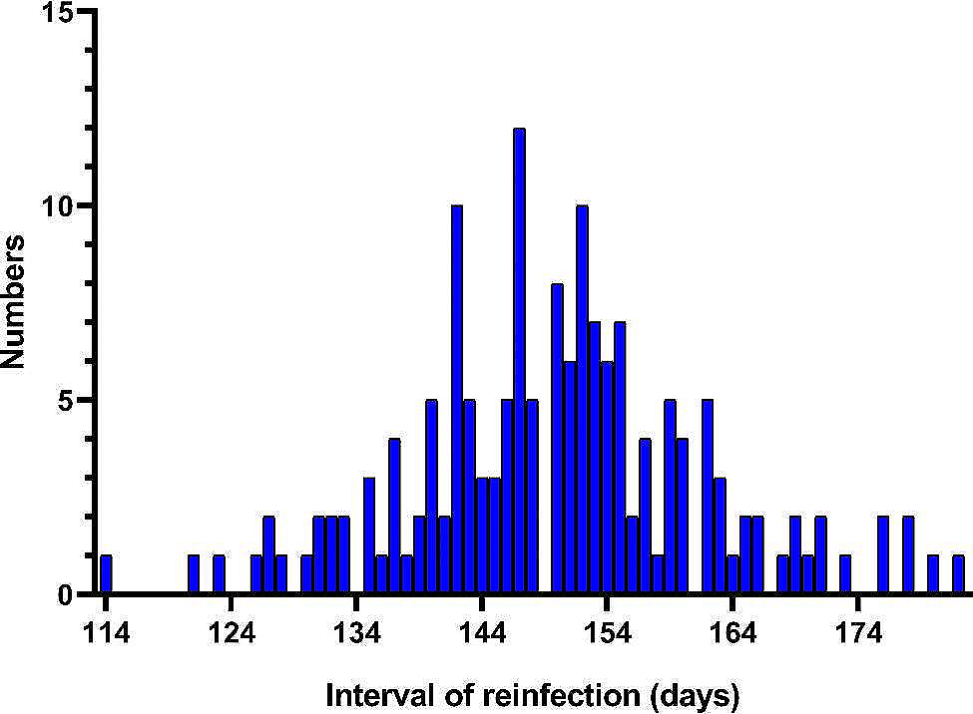
The interval of SARS-CoV-2 reinfection in HCWs

**Fig. 5:**
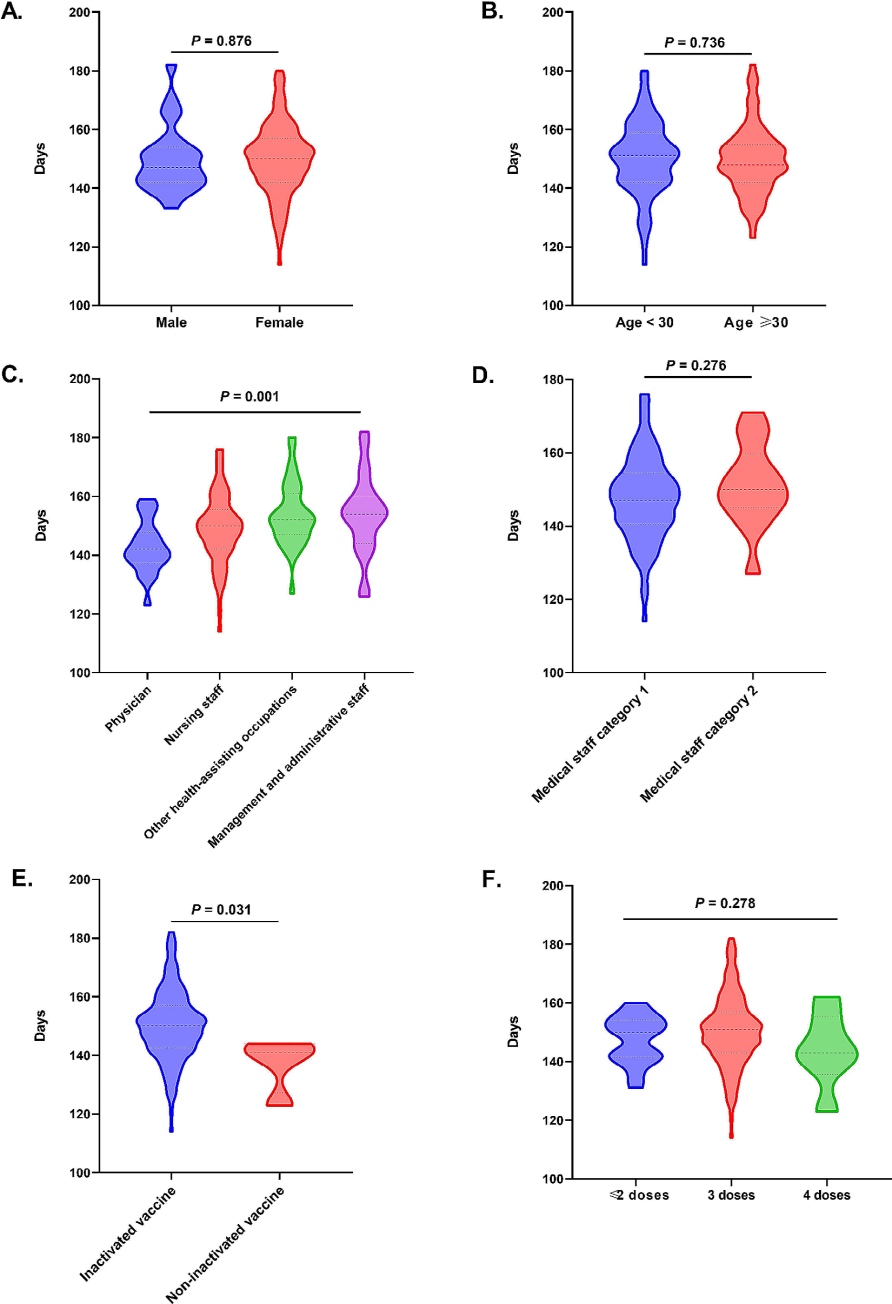
The average interval of SARS-CoV-2 reinfection in HCWs. **A**. The average interval of SARS-CoV-2 reinfection among male and female HCWs. **B**. The average interval of SARS-CoV-2 reinfection among HCWs aged < 30 years and ≥ 30 years. **C**. The average interval of SARS-CoV-2 reinfection among HCWs with different types of employment. **D**. The average interval of SARS-CoV-2 reinfection among HCWs with two medical staff category. **E**. The average interval of SARS-CoV-2 reinfection among HCWs received inactivated vaccine and non-inactivated vaccine. **F**. The average interval of SARS-CoV-2 reinfection among HCWs received ≤ 2 doses, 3 doses, and 4 doses of vaccination

The interval of SARS-CoV-2 reinfection in 161 HCWs ranged from 114 to 182 days, with a mean (SD) interval of 149.9 (11.9) days (Fig. 4). There was no statistical difference in reinfection intervals between different gender and age groups (all *P* >0.05), but there was a statistical difference among different types of employment (*P* < 0.05) (Fig. 5A ∼ 5C). Physicians showed the shortest mean (SD) interval of 142.8 (8.8) days, while management and administrative staff had the longest mean (SD) interval of 153.8 (13.5) days (Fig. 5C). The medical staff category1 had the shorter mean (SD) interval of 147.6 (11.5) days than the medical staff category 2 that of 151.2(11.5) days, but there was no statistical difference between two medical staff category (*P*>0.05) (Fig. 5D). The infection interval of non-inactivated vaccines group was shorter than that of inactivated vaccines group [150.3 (11.9) days vs. 137.3 (9.6) days, *P* < 0.05) (Fig. 5E). However, this may be related to the inactivated vaccine vaccination in China, as only 4 of the re-infected individuals had received a non-inactivated vaccine. There was no statistical difference between the groups that received ≤ 2 doses, 3 doses, and 4 doses of vaccination (*P*>0.05) (Fig. 5F).

## Discussion

In this study, we reported the trend of first infection and reinfection of SARS-CoV-2 in HCWs from April to June 2023. The reinfection rate among HCWs was 11.5%. Of these HCWs, 80.9% of the infected ones were female (76.2% for first infection vs. 83.9% for reinfection), Moreover, the highest proportion of SARS-CoV-2 infection, particularly the reinfection population, was observed in nursing staff. Furthermore, 98.9% of HCWs showed symptoms of the infection. But the symptoms of reinfection were milder and displayed fewer systemic symptoms than those of the first infection. In terms of the interval between the first infection and the reinfection, only 1.2% of reinfection cases occurred at intervals of no less than 180 days, and physicians had the shortest mean (SD) interval of 142.8 (8.8) days. These results emphasize the importance of ongoing optimization of strategies to prevent COVID-19, especially in the era of Omicron.

As a teaching hospital, it had a median age of HCWs of 31 years (P25, P75: 27, 35). This indicates that the age of HCWs in this study is lower compared to previously reported data [35, 36]. It is also relevant to note that the younger HCWs are more susceptible to infection and reinfection compared to relatively older ones in Omicron wave [37]. Previous studies have reported that females are more susceptible to COVID-19 [12, 38] and have a higher risk of reinfection [23]. In the results of a previous study, most of cases are nurses (48%), followed by physicians (25%) and other HCWs (23%) [39], which are similar to the results of our study. It may be put down to the reason that nurses typically spend a significant amount of time providing direct patient care. These indicate that nurses are at a higher risk of COVID-19 infection. Therefore, it is crucial to implement protective policies to reduce the transmission of SARS-CoV-2 in hospitals.

HCWs with Omicron infection have a high rate of symptom presentation. Earlier studies have reported that 3%∼23% of Omicron infection are asymptomatic [17, 40–42]. However, we discovered that only 1.2% of HCWs in this study were asymptomatic. As revealed by previous studies, the predominant symptoms among 1520 healthcare personnel infected with the Omicron variant in America (2021–2022) were pharyngitis (65%), cough (62%), fatigue (53%), headache (53%), rhinorrhea (51%), and myalgia (47%) [41]; while in China (2022–2023), the most frequent symptoms of 932 patients infected with the Omicron variant were fever (91%), cough (84%), weakness (77%), headache and dizziness (76%), and myalgia (74%)) [18]. Further, a systematic review and meta-analysis reported that the most common symptoms during the first infection and reinfection were fever (41.1%), cough (35.7% and 44.6%), myalgia (34.5% and 33.3%), fatigue (23.8% and 25.6%), and headaches (24.4% and 21.4%) [30]. In this study, we found that the main symptoms during the first infection and reinfection in this Omicron wave were sore throat (78.2% and 77.0%), fever (83.2% and 50.9%), fatigue (64.4% and 59.0%), dry cough (61.4% and 60.3%), nasal obstruction (58.4% and 55.3%), rhinorrhea (46.5% and 60.9%), and myalgia (56.4% and 37.9%). In addition, 63.4% of Omicron-infected HCWs had a fever. This percentage is much higher than that reported by the early studies (32%∼41.1%) [17, 30, 40, 41], but it is significantly lower than the data reported during the rapid spread of Omicron in China (91%) [18]. In this study, as rhinorrhea occurred more prominently in reinfection than in first infection, even when other symptoms are fewer in reinfection than in first infection, we can consider the following perspectives: (1) Differential immune memory response: Upon reinfection, the human immune system retains memory of the pathogen and may produce a more rapid and specific immune response against it. This could lead to certain immune-mediated symptoms, such as rhinorrhea, being more pronounced compared to the initial infection. (2) Variation in inflammatory responses: Re-infection might trigger an inflammatory response pattern different from the initial infection, resulting in more prominent local symptoms like rhinorrhea, while systemic symptoms are reduced due to immunomodulatory effects. (3) Changes in pathophysiological mechanisms: The pathophysiological processes of each infection might differ, potentially related to variations in the virulence of the pathogen, changes in the host’s immune status, or other factors, which could affect symptom presentation [31]. Although the infection cases among this particular group of young HCWs did not required hospital admissions, considering the symptoms caused by Omicron in first infection and reinfection, it is necessary to take precautions to mitigate the impact of the infection as well as to address the shortage of labor force.

COVID-19 vaccines have been documented to be able to reduce the risk of reinfection with SARS-CoV-2 [43, 44]. On the other hand, the effectiveness of the first infection against reinfection declines over time since first infection [45]. More importantly, protection offered by both vaccination and previous infection are not completely effective against SARS-CoV-2 reinfection [23]. Ana Rubia Guedes et al. found that the mean interval between infections in days (range) is 507 (122–674) days (approximately 16 months) in Omicron period during 1 January and 10 March, 2022 [27]. With the Omicron strain evolving, the protection conferred by previous infection against reinfection waned faster over time, resulting in a shorter interval between infections [46]. In the present study, the interval for SARS-CoV-2 reinfection in days (range) was 149.9 (114–182) days (approximately 5 months), which is shorter than previous data. A study of 173 COVID-19 patients found that the antibody titers in critically ill patients are significantly higher than those in non-critically ill patients [47]. Many people infected with SARS-CoV-2 remain asymptomatic, so their antibody titers may not have increased or may have lasted for a very short time. Viruses that cause local infections, such as viruses on mucosal surfaces and those without systemic viremia (such as influenza virus, respiratory syncytial virus, seasonal coronavirus), elicit weaker responses, and antibody titers last for a shorter duration. Additionally, the mutation of new coronavirus strains and the extension of vaccination intervals led to a relatively shorter interval between reinfections in this study compared to other studies. In addition, physicians and nursing staffs, expcially who from the clinical departments had the shorter interval. Medical institutions should still keep vigilant and implement protective measures.

In this study, the reinfection rate among HCWs was 11.5%. This is similar to previous studies [22, 23]. The reasons for COVID-19 reinfection are multifaceted, primarily including low antibody levels, the shortened duration of immune protection provided by antibodies, the mutation of viral strains, non-adherence to epidemic prevention measures by the population, hesitancy towards vaccination, and public fatigue due to the prolonged pandemic. There is a need for ongoing vigilance without assuming protection after the first episode [47]: (1) Strict adherence to COVID-19 appropriate behavior and other precautions is key to the long-term management of the pandemic. This includes maintaining correct wearing of personal protective equipment, strictly adhering to hand hygiene standards, maintaining good respiratory hygiene practices, enhancing personal health monitoring, following infection control measures, and strengthening individual immunity, among others. (2) Get vaccinated promptly with the latest vaccine based on the mutated virus and Variants of Concern, even after an infection has occurred. (3) During the pandemic, be aware of community transmission, and when a family member becomes infected, avoid close contact with them to minimize the risk of healthcare workers bringing community infections into the hospital.

Undoubtedly, this study has some strengths. First of all, its participants were HCWs. Secondly, the symptoms of first infection and reinfection were compared in this Omicron wave. Besides, this study disclosed the reinfected interval with different Omicron sublineages. To some extent, the final results provide guidance for HCWs in the prevention and management of the SARS-CoV-2 epidemic. However, several limitations still exist. Firstly, symptoms may be not comprehensively collected due to the questionnaire’s design, such as the severity of symptoms or the number of days individuals were affected by the symptoms during each infection. Secondly, due to the limitations of the detection conditions, the participants who tested positive for SARS-CoV-2 were not further identified by lineage with RT-PCR. Based on the data released by China CDC, the Omicron variant may be assumed to be dominant at the time of the study. Thirdly, the preventive health practice of HCWs is an important factor that can contribute to reducing infections in HCWs. However, we did not collect data on certain factors related to HCWs, such as mask wearing, hand washing, and the use of personal protective equipment (PPE) during procedures.

## Conclusion

The reinfection rate among HCWs was 11.5%. The main symptoms during the first infection in this Omicron wave were fever and sore throat, while sore throat was the main symptom in the reinfection; the average interval for SARS-CoV-2 reinfection was 149.91 (range: 114–182, SD = 11.94) days. Therefore, it is necessary to manage the SARS-CoV-2 pandemic among HCWs and provide essential health services. The healthcare workforce could take the required steps to prevent SARS-CoV-2 infection by maintaining SARS-CoV-2 surveillance, receiving booster doses of vaccination, and strengthening personal protection to decrease occupational exposure to the virus. Additionally, institutions that offer support should establish early warning and intervention strategies based on the frequency of SARS-CoV-2 reinfection.

### Electronic supplementary material

Below is the link to the electronic supplementary material.

Supplementary Material 1

## Data Availability

The data sets supporting the results of this article are included within the article. All data generated or used during the study are available from the corresponding author by request.

## Abbreviations

HCWs: Healthcare workers
SARS-CoV‐2: Severe acute respiratory syndrome coronavirus 2
COVID-19: Coronavirus disease 2019
WHO: World Health Organization
VOCs: Variants of concern
VOIs: Variants of interest
CDC: Center for Disease Control and Prevention
RT-PCR: Reverse transcription– polymerase chain reaction
Ct: Cycle threshold
SPSS: Statistical Product and Service Solutions
PPE: Personal protective equipment
