# Supplementary material for: The symptoms and interval of Omicron SARS-CoV-2 reinfection among healthcare workers in a hospital of Southern China: a cross-sectional study
Source: BMC Infect Dis. 2024 Mar 27;24:354. doi: 10.1186/s12879-024-09221-3 (PMC10967136; doi:10.1186/s12879-024-09221-3)
Supplement: Supplementary file 1 — Supplementary Material 1 [file 12879_2024_9221_MOESM1_ESM.docx]

Table A.1 Questionnaires from HCWs infected with SARS-CoV-2

Name: Sex: □Female □Male Age: (years)

Department:

Type of employment: □ Physician

□ Nursing staff

□ Other health-assisting occupations

(medical technicians or pharmacists)

□ Management and administrative staff

Q1: Your infection date:

Q2: Have did you confirm your infection with SARS-CoV-2? ( Please upload photo evidence)

□ RT-PCR, the first test date:

□ Antigen kit test, the first test date:

□ No

Q3: Have you experienced a reinfection with SARS-CoV-2?

□ Yes, the first infeciton date: □ No

Q4: What symptoms of COVID-19 did you experience?

□I have no symptoms.

□Fever (≥37.3℃); □dry cough; □nasal obstruction; □rhinorrhea; □sore throat; □diarrhea; □fatigue; □myalgia; □conjunctivitis; □anosmia or dysgeusia;

□Others :

Q5: Have you been hospitalized for this infection?

□ Yes □ No
